# The diagnostic value of histogram analysis of DWI and DKI for the mismatch repair status of rectal adenocarcinoma

**DOI:** 10.1016/j.heliyon.2024.e37526

**Published:** 2024-09-05

**Authors:** Hao Chen, Zhicheng Jin, Xiaoxiao Dai, Juan Zhu, Guangqiang Chen

**Affiliations:** a Department of Medical Imaging, Anqing Municipal Hospital, Anqing, China; b Department of Radiology, The Second Affiliated Hospital of Soochow University, Suzhou, China; c Department of Nuclear Medicine, The Second Affiliated Hospital of Soochow University, Suzhou, China; d Department of Pathology, The Second Affiliated Hospital of Soochow University, Suzhou, China

**Keywords:** Rectal adenocarcinoma, Magnetic resonance imaging, Diffusion weighted imaging, Mismatch repair status, Histogram analysis

## Abstract

**Objectives:**

To compare the diagnostic value of histogram analysis derived from diffusion weighted imaging (DWI) and diffusion kurtosis imaging (DKI) in differentiating the mismatch repair (MMR) status of rectal adenocarcinoma.

**Methods:**

DWI and DKI were performed in 124 patients with rectal adenocarcinoma, which were divided into deficient mismatch repair (dMMR) group and proficient mismatch repair (pMMR) group. The patients' general clinical information, pathology and image characteristics were compared. The histogram analysis of apparent diffusion coefficient (ADC), diffusion kurtosis (K) and diffusion coefficient (D)derived from DWI and DKI at b values of 1000 and 2000 s/mm^2^ were calculated. The diagnostic efficacy of quantitative parameters for MMR in rectal adenocarcinoma was compared.

**Results:**

The mean, 50th, 75th and 90th in ADC quantitative parameters of dMMR group were lower when the b value was 2000 s/mm^2^ (all *P* < 0.05). With b value of 1000 s/mm^2^, the 10th, 25th, and 50th in the dMMR group were lower, and the skewness was higher (all *P* < 0.05). D values (10th, 25th and 50th) derived from DKI quantitative parameters were lower in the dMMR group. The K values (75th, 90th and Kskewness) were higher in the dMMR group, while Kkurtosis was lower (all *P* < 0.05). The results of multivariate logistic regression analysis showed that ADC75th(b = 2000 s/mm^2^), ADCskewness (b = 1000 s/mm^2^) and Kskewness were the statistical significant parameters (*P* = 0.014, 0.036 and 0.002, respectively), and the AUC values were 0.713, 0.818 and 0.835, respectively.

**Conclusion:**

Histogram analysis derived from DWI and DKI can be good predictor of MMR. Kskewness is the strongest independent factor for predicting MMR.

## 1 Background

Rectal adenocarcinoma is a common gastrointestinal malignant tumor that is among the tumours with the greatest global growth rates in terms of morbidity and death [1,2]. The survival rates of patients with rectal adenocarcinoma have increased in recent years, largely due to advancements in diagnostic techniques, surgical procedures, and the introduction of novel therapeutic options [3]. In particular, immune checkpoint therapy (ICT) represents a significant advancement in the treatment of rectal adenocarcinoma [[4], [5], [6], [7]]. Mutations and methylation of DNA mismatch repair proteins lead to high levels of satellite instability in rectal adenocarcinoma with clear guidance in the prediction of occurrence and prognosis [[8], [9], [10], [11]]. In 2017, the FDA approved ICT for the treatment of high mutation burden tumours in deficient mismatch repair (dMMR) or microsatellite instability-high (MSI-H). However, no significant benefit was observed for tumours in proficient mismatch repair (pMMR) or microsatellite instability-low (MSI-L) [12,13]. Therefore, accurate diagnosis mismatch repair (MMR) status of rectal adenocarcinoma is critical for treatment selection as well as for reducing recurrence rates and improving long-term survival.

Currently, there are three main detection methods for MMR, including polymerase chain reaction (PCR), immunohistochemistry (IHC) and Next-generation sequencing (NGS) [14]. Nevertheless, dMMR is identified in only approximately 15 % of rectal adenocarcinomas, with the remaining 85 % exhibiting pMMR [15]. Additionally, IHC is associated with the drawback of being time-consuming and labor-intensive. Moreover, tumor heterogeneity cannot be detected dynamically, expensive and invasive puncture are all limitations of pCR, NGS and IHC. Prognostic differences based on tumor aggressiveness and evolving treatment paradigms emphasize the need for non-invasive tools, dynamic monitoring to accurately identify the MMR of rectal adenocarcinoma in the quest for more accurate and personalized treatment options.

Previous studies have demonstrated that the differential diagnosis of benign and malignant tumours can be achieved through the use of whole tumour histogram analysis, which is based on magnetic resonance diffusion weighted imaging (DWI) and diffusion kurtosis imaging (DKI) [[16], [17], [18]]. Furthermore, it is more effective than conventional sequences in extracting the biological characteristics of tumours, thereby enabling the prediction of the expression differences of tumor genes, protein molecules, and the tumor microenvironment [[19], [20], [21], [22]]. Moreover, DKI quantitative parameters are valuable in preoperative identification of rectal adenocarcinoma with expression of Ki-67 and Her-2 [23,24]. Prior research has indicated that T1WI, T2WI, and DCE parameters play a limited role in distinguishing MMR in rectal cancer. ADC derived from conventional DWI quantifies diffusion behavior of water molecule following Gaussian distribution. However, it can be contaminated by non-Gaussian signal at higher b-values, leading to large variation and poor performance. In contrast, DKI separates Gaussian and non-Gaussian diffusion effect, with MD representing diffusion and MK quantifying tissue complexity. Therefore, MD and MK from DKI could effectively reflect cellularity (MD) and tissue complexity (MK) in rectal adenocarcinoma.

Histogram analysis is widely used in radiological studies as it can visually analyse the distribution of data. Previous studies have demonstrated that whole lesion volume histogram analysis can be used for the differential diagnosis of benign and malignant tumours in different systems, as well as the staging and grading of malignant tumours. In addition, it can also be able to comprehensively reflect the heterogeneity of the tumour and the invasiveness of the various parts within the tumour, more effectively and comprehensively extract the biological characteristics of the tumour and reflect the microstructure of the tumour. However, to the best of our knowledge, there is no clear evidence of a relationship between quantitative parameters from DWI and DKI with MMR of rectal adenocarcinoma. Consequently, the objective of this study was to determine whether these parameters could be employed for the preoperative diagnosis of MMR, which would subsequently serve as a foundation for the individualised treatment of clinical rectal adenocarcinoma patients.

## 2 Materials and methods

### 2.1 Patients

**Fig. 1:**
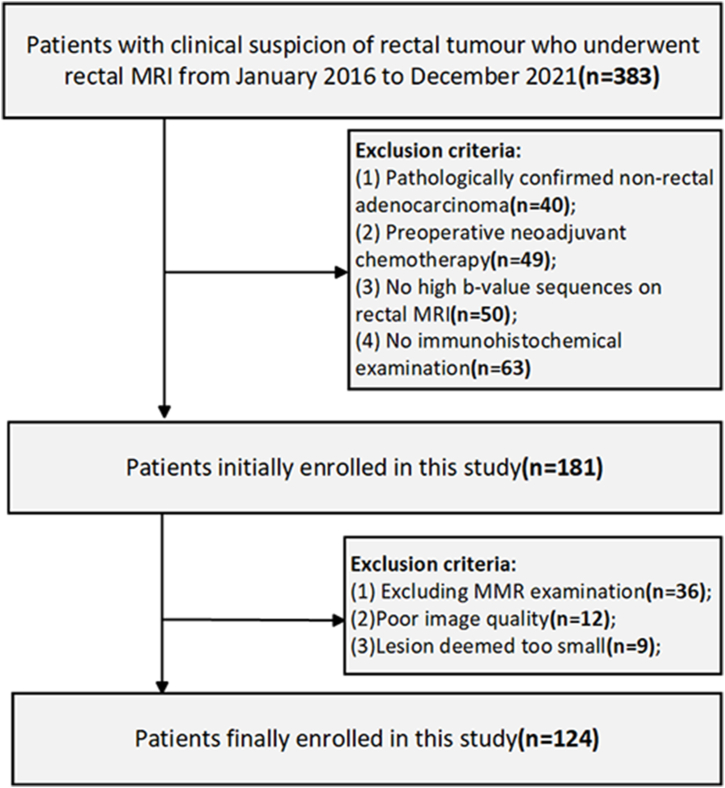
Flow chart of study participants.

The study was approved by the local ethics committee. 383 patients with clinical suspicion of rectal tumor and underwent rectal MRI from January 2016 to December 2021 were analyzed retrospectively. Baseline data were collected from the patients during the preoperative week, including gender, age, serum carbohydrate antigen 19-9 (CA19-9; normal level:<30.00 U/mL), serum carcino-embryonic antigen (CEA; normal level:<5.00 ng/ml). Similarly, postoperative pathological and immunohistochemical data were collected, which including the tumor grade, pathological T-stage (pT), pathological N-stage (pN), presence of vascular invasion (pVI) and nerve invasion (pNI). The inclusion criteria for this study were as following: Patients were confirmed with rectal cancer by endoscopic biopsy or postoperative pathology; No preoperative neoadjuvant therapy before the rectal MRI examination; Routine preoperative rectal MRI, including DWI(b value of 1000 and 2000 s/mm^2^) and DKI scans; Routine pathology and IHC were performed on postoperative specimens. The exclusion criteria were as follows: postoperative tumor specimens not examined for MMR; Poor image quality of the rectal MRI examination; The smaller size of the rectal lesion made it difficult to delineate the area of interest (ROI). The flow chart is shown in Fig. 1.

### 2.2 Image data acquisition

The patients were emptied the bladder before rectal MRI examination. The research machine was a Philips 3.0T Ingenia superconducting MRI scanner. The main scanning sequence is as follows: Sagittal fast spin echo T2WI (FSE-T2WI), Fat suppression sequence T2WI (T2WI-SPAIR), regular axial T1WI, oblique axial High Resolution-T2WI (HR-T2WI; the location line was perpendicular to the intestinal canal in which the lesion is located), oblique axial DWI sequences (b-values of 0, 500, 1000 and 2000 s/mm^2^, respectively) and DKI sequences (b-values of 0, 200, 500, 1000, 1500 and 2000 s/mm^2^, respectively). The specific scanning parameters are shown in Table 1.

**Table 1:** Scanning parameters of diffusion sequences.

Sequence	T1WI	T2WI	T2WI-SPAIR	HR-T2WI	DWI	DKI
TR (ms)	571	4156	4578	4228	4500	6000
TE (ms)	8	85	70	85	65	80
NSA	1	2	2	1	2	2
Matrix	276 × 406	300 × 300	168 × 215	300 × 289	104 × 130	104 × 130
View (mm)	400	240	400	180	260	260
Thickness (mm)	6	3	6	3	3	3
Slice gap (mm)	1	1	1	0	0	0
Time (min)	1:11	3:44	1:50	4:26	3:36	5:23

TR, repetition time; TE, echo time; NSA, Number of Signals Averaged; T2WI, T2-weighted imaging; T1WI, T1-weighted imaging; T2WI-SPAIR, T2-weighted imaging SPectral Attenuated Inversion Recovery; HR-T2WI, High Resolution T2-weighted imaging; DWI, diffusion-weighted imaging.

### 2.3 Image data analysis and processing

Two radiologists with more than three and five years of diagnostic experience performed MRI image post-processing without knowledge of the patient's unique pathological staging or immunohistochemistry data. The MRI images of rectal adenocarcinoma were interpreted and tumor location, T Stage on MRI (mrT), N Stage on MRI (mrN), circumferential resection margin on MRI (mrCRM) and extramural vascular invasion on MRI (mrEMVI) were evaluated according to the international diagnostic protocols for magnetic resonance TNM staging of rectal cancer [25]. When two radiologists could not reach consensus, an experienced radiologist with 25 years of experience was further consulted for a final opinion.

**Fig. 2:**
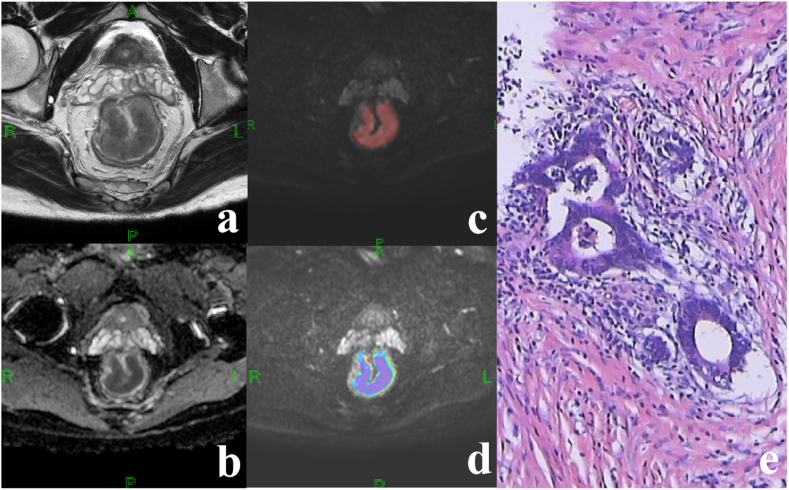
Middle-differentiated adenocarcinoma of the middle-upper rectal in a 48-year-old male patient. Immunohistochemistry results indicated dMMR status: MLH1 (+), PMS2(−), MSH2(+), MSH6(+). (a) HR-T2WI showed significant thickening of the proximal 3/4 of the rectal wall with slightly low signal. (b) ADC map of the lesion. (c) ROI of the lesion on DWI when b = 1000 s/mm2. (d) Corresponding pseudo-color map of lesions with ADCmean value of 0.877 × 10-3 mm^2^/s. (e) Fibrous tissue hyperplasia with lymphocytic infiltration around localized cancerous tissue (HE: × 100).

**Fig. 3:**
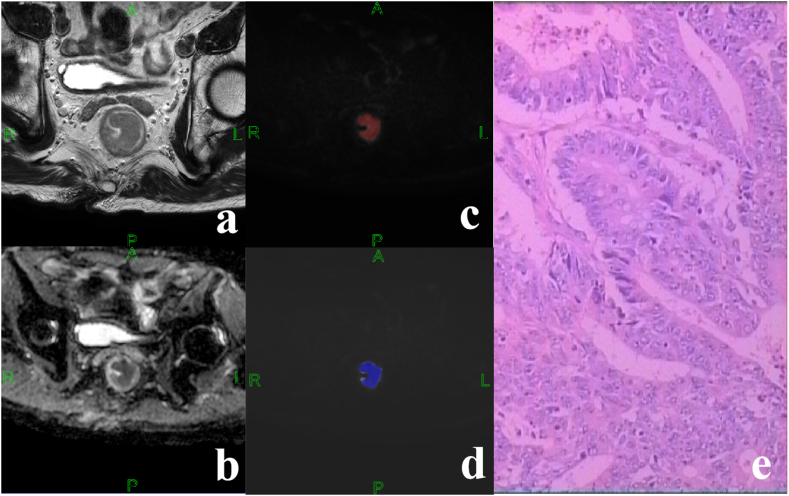
Middle-differentiated adenocarcinoma of the middle rectal in a 71-year-old male patient. Immunohistochemistry results indicated pMMR status: MLH1 (+), PMS2(+), MSH2(+), MSH6(+). (a) HR-T2WI showed significant thickening of the proximal 3/4 of the rectal wall with slightly low signal. (b) ADC map of the lesion. (c) ROI of the lesion on DWI when b = 2000 s/mm2. (d) Corresponding pseudo-color map of lesions with ADCmean value of 1.183 × 10-3 mm^2^/s. (e) No significant lymphocytic infiltration (HE: × 100).

MRI quantitative parameters were extracted using FireVoxel software. Two radiologists combined HR-T2WI images and post-processed ADC images, avoiding intestinal contents and hemorrhagic necrotic areas, and outlining layer by layer along the edge of the tumor to obtain the ROI of the whole tumor on the DWI images with b-value of 1000 and 2000 s/mm^2^, respectively. The ADC values were calculated based on the diffusion-weighted imaging single-exponential model Sb/S0 = exp (-b*ADC) [26], and the pseudo-color maps were generated (Fig. 2). Considering that there is a slight mismatch in the location of the lesion on the image due to the involuntary movement of the gastrointestinal tract during MRI scanning, so the radiologist should make sure that the location of the ROI match to the location on the HR-T2WI image. ROIs were manually outlined layer by layer along the tumour margins on the DKI images at b = 1000 s/mm2, respectively. The diffusion coefficient (D value) and kurtosis coefficient (K value) were measured according to the diffusion kurtosis model Sb/S0 = exp (-b*D+ b^2*D^2*K/6) (Fig. 3) [27]. A consistency test was performed on the two physicians, and if the consistency was good, the average of the measured parameters of the two physicians was used as the final data (ICC>0.80). The DWI and DKI histogram parameters (mean, 10th, 25th, 75th, 90th, skewness and kurtosis) were obtained using MATLAB 2018b and SPSS 22.0 software.

### 2.4 Pathology and immunohistochemistry

**Fig. 4:**
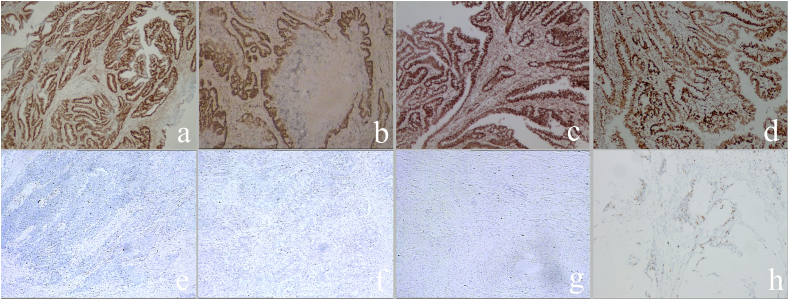
Immunohistochemical staining results for MMR protein in rectal adenocarcinoma: MLH1 (a, e), PMS2(b, f), MSH2(c, g) and MSH6(d, h). Brown staining of tumor cell nuclei was defined as positive expression of MMR protein (a-d), and the reverse was defined as negative expression (e-h).

Pathologic staging of rectal adenocarcinoma was based on the 8th edition of the TNM staging criteria for tumours designated by the American joint committee on cancer (AJCC) [28]. Two pathologists with 5 and 8 years of diagnostic experience evaluated rectal adenocarcinoma with DNA mismatch repair proteins (MLH1, PMS2, MSH2, and MSH6). The pathologists were unaware of the patient's imaging and clinical information. Criteria for MMR in rectal adenocarcinoma: dMMR is negative staining for at least one of the four MMR proteins; pMMR is positive staining for all four MMR proteins (Fig. 4).

### 2.5 Statistical analysis

Statistical analysis was performed with the software SPSS 22.0 and Medcalc 12.1. Shapiro-Wilk test was used for normality test of the measurements data. The data that conformed to normal distribution and satisfied variance chi-square were analyzed using independent samples *t*-test and expressed as mean ± standard deviation. Non-normally distributed data were analyzed by Mann-Whitney *U* test and expressed using the median (first quartile, third quartile). Count data were analyzed by chi-square test. The diagnostic efficacy of different parameters was evaluated by area under the curve (AUC), cutoff, sensitivity, specificity and 95 % confidence interval. *P* < 0.05 indicates statistically significant difference. For statistically significant parameters were enrolled into multivariate logistic regression analysis to further explore the associations between quantitative MRI parameters and MMR of rectal adenocarcinoma.

## 3 Results

### 3.1 Patient characteristics

A total of 124 patients with rectal adenocarcinoma were included in this study, of which 29 were in the dMMR group and 95 were in the pMMR group. The general clinical information of patients with rectal adenocarcinoma is summarized in Table 2. There were no statistically differences in gender, age, tumor location, tumor grade, pT, pNI and preoperative CA19-9 levels between the dMMR and pMMR groups (all *P* > 0.05). The pN, pVI and preoperative CEA levels were significantly different between the dMMR and pMMR groups (*P* < 0.001, <0.001 and 0.006, respectively), in which the pathological metastatic lymph nodes and the vascular invasion in the dMMR group were lower than the pMMR group, and the preoperative serum CEA level in the dMMR group was significantly higher than the pMMR group. There were no statistically differences in tumor location, mrT, mrN, mrCRM and mrEMVI between the dMMR and pMMR groups in patients with rectal adenocarcinoma (all *P* > 0.05).

**Table 2:** Demographic and clinical characteristics of the patients with rectal adenocarcinoma.

Characteristics	dMMR (n = 29)	pMMR (n = 95)	P value
**Gender**			0.087
Male	23 (79.3 %)	59 (62.1 %)	
Female	6 (20.7 %)	36 (37.9 %)	
**Age**	62.93 ± 11.10	61.32 ± 10.36	0.611
**CEA**			**0.006**
<5 ng/ml	13 (44.8 %)	69 (76.8 %)	
≥5 ng/ml	16 (55.2 %)	26 (23.2 %)	
**CA19-9**			0.266
<30 U/ml	20 (69.0 %)	75 (80.4 %)	
≥30 U/ml	9 (31.0 %)	20 (19.6 %)	
**Tumor location**			0.292
High	4 (14.3 %)	23 (24.2 %)	
Middle	19 (64.3 %)	47 (49.5 %)	
Low	6 (21.4 %)	25 (26.3 %)	
**Tumor grade**			0.510
High	4 (13.8 %)	7 (7.3 %)	
Middle	18 (62.1 %)	59 (62.1 %)	
Low	7 (24.1 %)	29 (30.5 %)	
**pT**			0.515
T1-2	12 (41.4 %)	33 (34.7 %)	
T3-4	17 (58.6 %)	62 (65.3 %)	
**pN**			**< 0.001**
N0	25 (86.2 %)	42 (44.2 %)	
N1-2	4 (13.8 %)	53 (55.8 %)	
**pVI**			**< 0.001**
–	27 (93.1 %)	55 (57.9 %)	
+	2 (6.9 %)	40 (42.1 %)	
**pNI**			0.060
–	24 (89.7 %)	61 (64.2 %)	
+	5 (17.2 %)	34 (35.8 %)	
**mrT**			0.432
T1-2	5 (17.2 %)	23 (24.2 %)	
T3-4	24 (89.7 %)	72 (75.8 %)	
**mrN**			0.596
N0	8 (27.6 %)	22 (23.2 %)	
N1	13 (44.8 %)	37 (38.9 %)	
N2	8 (27.6 %)	36 (37.9 %)	
**mrCRM**			0.982
–	21 (72.4 %)	69 (72.6 %)	
+	8 (27.6 %)	26 (27.4 %)	
**mrEMVI**			0.059
–	18 (62.1 %)	40 (42.1 %)	
+	11 (37.9 %)	55 (57.9 %)	

dMMR, deficient mismatch repair; pMMR, proficient mismatch repair; CEA, Carcinoembryonic antigen; CA19-9, Carbohydrate antigen 19–9; pT, T Stage on pathology; pN, N Stage on pathology; pVI, vascular invasion on pathology; pNI, neurological invasion on pathology; mrT, T Stage on MRI; mrN, N Stage on MRI; mrCRM, Circumferential resection margin on MRI; mrEMVI, Extramural vascular invasion on MRI.

### 3.2 Histogram index analysis of quantitative parameters of DWI and DKI

The histogram analysis parameters of DWI and DKI measured by two radiologists were in good consistency, with ICC values greater than 0.80 (ranging from 0.858 to 0.945 and 0.898–0.951, respectively), detailed results as shown in Table 3, Table 4.

**Table 3:** Comparison of DWI quantitative parameter histogram analysis between the deficient mismatch repair group and the proficient mismatch repair group.

Parameters	dMMR (n = 29)	pMMR (n = 95)	ICC (95%CI)	Z	P value
(b = **2000** s/mm^2^)					
ADCmean	0.889 ± 0.075	0.985 ± 0.133	0.945 (0.885–0.974)	−3.563	**0.001**
ADC10th	0.549 ± 0.148	0.614 ± 0.097	0.898 (0.846–0.933)	−1.204	0.549
ADC25th	0.698 ± 0.069	0.746 ± 0.093	0.935 (0.900–0.958)	−1.794	0.077
ADC50th	0.836 ± 0.079	0.919 ± 0.118	0.932 (0.814–0.968)	−3.147	**0.004**
ADC75th	1.045 ± 0.111	1.162 ± 0.176	0.932 (0.814–0.968)	−2.369	**0.021**
ADC90th	1.315 ± 0.129	1.461 ± 0.253	0.930 (0.875–0.959)	−3.011	**0.004**
skewness	0.884 ± 0.444	0.958 ± 0.428	0.888 (0.796–0.935)	−0.496	0.621
kurtosis	1.111 ± 1.574	1.355 ± 1.310	0.919 (0.827–0.957)	0.635	0.527
(b = **1000** s/mm^2^)					
ADCmean	1.043 ± 0.140	1.029 ± 0.131	0.875 (0.799–0.922)	0.333	0.740
ADC10th	0.529 ± 0.201	0.657 ± 0.126	0.858 (0.772–0.912)	−2.614	**0.004**
ADC25th	0.715 ± 0.095	0.815 ± 0.104	0.872 (0.792–0.920)	−3.264	**0.002**
ADC50th	0.917 ± 0.178	0.994 ± 0.124	0.892 (0.827–0.933)	−2.116	**0.038**
ADC75th	1.219 ± 0.217	1.223 ± 0.164	0.957 (0.931–0.955)	−0.066	0.947
ADC90th	1.545 ± 0.214	1.504 ± 0.224	0.929 (0.888–0.929)	0.631	0.530
skewness	2.224 ± 1.150	0.801 ± 0.125	0.935 (0.897–0.957)	3.851	**<0.001**
kurtosis	1.730 ± 1.128	2.141 ± 0.189	0.901 (0.840–0.938)	−0.780	0.438

dMMR, deficient mismatch repair; pMMR, proficient mismatch repair; ADC, Apparent diffusion coefficient ( × 10^−3^ mm^2^/s).

**Table 4:** Comparison of DKI quantitative parameter histogram analysis between the deficient mismatch repair group and the proficient mismatch repair group.

Parameters	dMMR (n = 29)	pMMR (n = 95)	ICC (95%CI)	Z	P value
Dmean	1.325 ± 0.170	1.403 ± 0.180	0.928 (0.876–0.956)	−1.451	**0.151**
D10th	0.642 ± 0.241	0.815 ± 0.211	0.934 (0.900–0.957)	−2.668	**0.010**
D25th	0.878 ± 0.153	1.033 ± 0.156	0.929 (0.888–0.955)	−3.355	**0.001**
D50th	1.162 ± 0.133	1.333 ± 0.186	0.951 (0.918–0.970)	−3.234	0.002
D75th	1.575 ± 0.225	1.703 ± 0.247	0.930 (0.894–0.955)	−1.756	0.084
D90th	2.124 ± 0.434	2.139 ± 0.335	0.927 (0.889–0.953)	−0.139	0.890
Dskewness	0.670 ± 0.548	0.685 ± 0.367	0.931 (0.895–0.955)	−0.098	0.923
Dkurtosis	0.444 ± 1.361	0.703 ± 1.111	0.928 (0.890–0.953)	−0.745	0.459
Kmean	0.960 ± 0.117	0.936 ± 0.107	0.909 (0.862–0.941)	0.749	0.456
K10th	0.624 ± 0.072	0.529 ± 0.326	0.912 (0.866–0.943)	1.070	0.288
K25th	0.809 ± 0.130	0.788 ± 0.172	0.930 (0.892–0.955)	0.429	0.669
K50th	1.003 ± 0.084	0.951 ± 0.113	0.908 (0.864–0.940)	1.607	0.133
K75th	1.169 ± 0.096	1.091 ± 0.145	0.902 (0.850–0.936)	1.920	**0.028**
K90th	1.417 ± 0.099	1.291 ± 0.132	0.898 (0.846–0.934)	3.360	**0.001**
Kskewness	0.111 ± 0.521	−0.672 ± 0.520	0.920 (0.878–0.948)	5.035	**<0.001**
Kkurtosis	2.489 ± 1.585	3.314 ± 1.700	0.914 (0.848–0.949)	−1.646	**0.027**

dMMR, deficient mismatch repair; pMMR, proficient mismatch repair; DKI, diffusion kurtosis imaging; D, apparent diffusion coefficients for non-Gaussian distributions( × 10^−3^ mm^2^/s); K, kurtosis coefficient( × 10^−3^ mm^2^/s).

When the b-value was 2000 s/mm^2^, the values of ADCmean, ADC50th, ADC75th and ADC90th in the dMMR group were lower than in the pMMR group (*P* = 0.001, 0.004, 0.021 and 0.004, respectively), with AUC values of 0.728, 0.711, 0.713, and 0.704, respectively. When the b value was 1000 s/mm^2^, the ADC10th, ADC25th and ADC50th in the dMMR group were lower than the pMMR group, and the skewness was higher than the pMMR group (*P* = 0.009, 0.002, 0.038, and <0.001, respectively), and the AUC were 0.727, 0.777, 0.681 and 0.818, the detailed results are shown in Table 3, Table 5.

**Table 5:** Diagnostic test characteristics of diffusion parameters between the deficient mismatch repair group and the proficient mismatch repair group.

Parameters	AUC(95%CI)	Cutoff	Youden index	Sensitivity	Specificity
**Clinical**					
CEA	0.670 (0.547–0.777)	5	0.339	57.14	76.70
pN	0.696 (0.575–0.801)	–	0.393	85.71	53.57
pVI	0.680 (0.556–0.797)	–	0.161	82.86	43.21
**b** = **1000**					
ADC10th	0.727 (0.607–0.827)	0.668	0.393	85.71	53.57
ADC25th	0.777 (0.661–0.868)	0.791	0.500	85.71	62.29
ADC50th	0.681 (0.559–0.787)	0.865	0.339	50.00	83.93
skewness	0.818 (0.745–0.893)	0.977	0.625	92.86	69.64
**b** = **2000**					
ADCmean	0.728 (0.609–0.828)	0.985	0.375	92.86	51.79
ADC50th	0.711 (0.590–0.813)	0.908	0.429	85.71	57.14
ADC75th	0.713 (0.623–0.814)	1.163	0.446	92.96	51.79
ADC90th	0.704 (0.583–0.807)	1.467	0.429	92.86	50.00
**DKI**					
D10th	0.687 (0.566–0.793)	0.779	0.412	85.71	55.36
D25th	0.773 (0.657–0.865)	1.005	0.518	92.86	58.93
D50th	0.808 (0.696–0.892)	1.204	0.661	85.71	80.36
K75th	0.712 (0.592–0.814)	1.110	0.446	92.86	51.79
K90th	0.788 (0.673–0.876)	1.397	0.536	71.43	82.14
Kskewness	0.835 (0.762–0.907)	−0.361	0.679	92.86	75.00
Kkurtosis	0.684 (0.562–0.790)	2.764	0.446	85.71	58.93

ADC, Apparent diffusion coefficient ( × 10^−3^ mm^2^/s); DKI, diffusion kurtosis imaging; D, apparent diffusion coefficients for non-Gaussian distributions( × 10^−3^ mm^2^/s); K, kurtosis coefficient ( × 10^−3^ mm^2^/s); AUC, Area under the curve; 95 % CI, 95 % confidence interval; CEA, carcinoembryonic antigen(ng/ml).

Among the quantitative DKI parameters, the D values (D10th, D25th, and D50th) of the dMMR group were lower than the pMMR group (*P* = 0.031, 0.001, and 0.002, respectively), with AUC of 0.687, 0.773, and 0.808, respectively. dMMR group's K values (K75th, K90th, and Kskewness) were all were higher than the pMMR group, and the K kurtosis was lower than the pMMR group (*P* = 0.014, 0.001, <0.001, 0.034, respectively), and the AUC were 0.712, 0.788, 0.835, and 0.684, respectively, and the detailed results are shown in Table 4, Table 5.

**Fig. 5:**
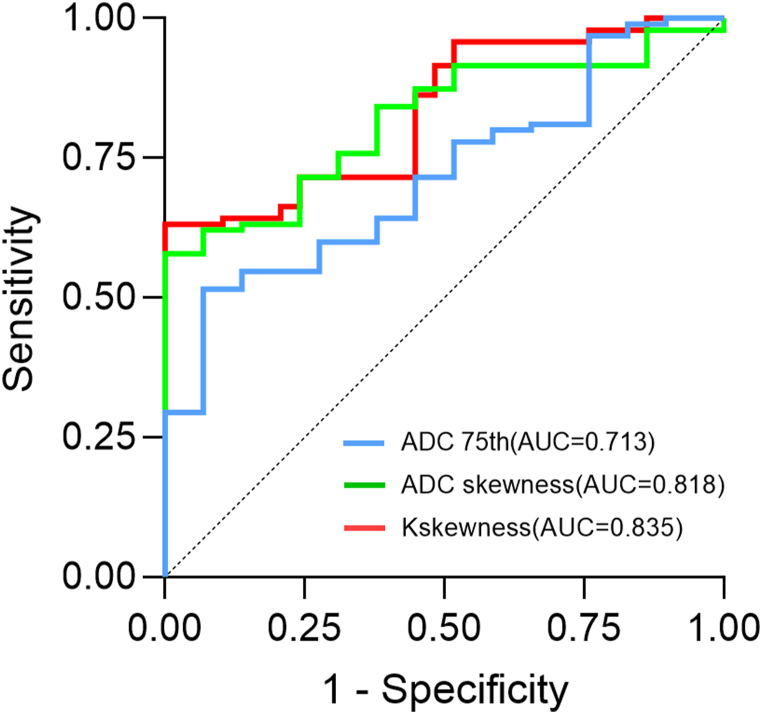
Receiver operating characteristic curves of ADC75th(b = 2000 s/mm^2^), ADCskewness (b = 1000 s/mm^2^) and Kskewness in the differential diagnosis of the mismatch repair (MMR) status of rectal adenocarcinoma.

The statistically significant DWI and DKI quantitative parameters were further screened by univariate regression analysis and multivariate logistic regression analysis, and the results showed that ADC75th(b = 2000 s/mm^2^), ADCskewness (b = 1000 s/mm^2^) and Kskewness were the statistical significant parameters (*P* = 0.014, 0.036 and 0.002, respectively). The specific results are shown in Table 6 and Fig. 5.

**Table 6:** Univariate and multivariate logistic regression analysis of quantitative MR parameters for identification MMR of rectal adenocarcinoma.

Parameters	Univariate regression analysis			Multifactor regression analysis		
	OR	95 % CI	p	OR	95 % CI	p
CEA	4.41	1.293, 15.042	**0.018**	N/A	N/A	0.139
pN	1.44	0.030, 0.706	**0.017**	N/A	N/A	0.287
pVI	1.32	0.142, 1.472	**0.026**	N/A	N/A	0.381
ADC10th(**b** = **1000**)	0.008	0.000, 0.344	**0.012**	N/A	N/A	0.228
ADC25th(**b** = **1000**)	0.006	0.000, 0.692	**0.006**	N/A	N/A	0.214
ADC50th(**b** = **1000**)	0.004	0.000, 0.853	**0.044**	N/A	N/A	0.778
skewness (**b** = **1000**)	2.226	1.307, 3.792	**0.003**	2.463	1.061, 5.718	**0.036**
ADCmean (**b** = **2000**)	0.004	0.000, 0.562	**0.016**	N/A	N/A	0.599
ADC50th(**b** = **2000**)	0.012	0.004, 0.713	**0.021**	N/A	N/A	0.907
ADC75th(**b** = **2000**)	0.016	0.008, 0.871	**0.025**	0.133	0.027, 0.666	**0.014**
ADC90th(**b** = **2000**)	0.003	0.000, 0.214	**0.040**	N/A	N/A	0.990
D10th	0.010	0.000, 0.417	**0.015**	N/A	N/A	0.976
D25th	0.001	0.000, 0.139	**0.005**	N/A	N/A	0.677
D50th	0.001	0.000, 0.124	**0.004**	N/A	N/A	0.353
K75th	23.243	0.927, 49.035	**0.053**	N/A	N/A	N/A
K90th	7.728	1.168, 45.596	**0.004**	N/A	N/A	0.293
Kskewness	17.694	3.037, 103.084	**0.001**	9.951	2.250, 44.018	**0.002**
Kkurtosis	0.729	0.495, 1.073	**0.109**	N/A	N/A	N/A

ADC, Apparent diffusion coefficient ( × 10^−3^ mm^2^/s); DKI, diffusion kurtosis imaging; D, apparent diffusion coefficients for non-Gaussian distributions( × 10^−3^ mm^2^/s); K, kurtosis coefficient ( × 10^−3^ mm^2^/s); AUC, Area under the curve; 95 % CI, 95 % confidence interval; CEA, carcinoembryonic antigen(ng/ml); OR, odds ratio.

## 4 Discussion

MMR status is nowadays acknowledged as an important factor in CRC that is used to guide decisions on the benefit of preoperative immunotherapy. However, there is no effective tool that enables noninvasive as well as dynamic detection the MMR of rectal adenocarcinoma. Rectal tumours of different MMR develop different biological heterogeneity, leading to differences in water diffusion. Therefore, DWI and DKI are useful biomarkers for predicting the heterogeneity of tumours. Based on this background, the conclusions drawn from our study suggest that quantitative parameters derived from DWI and DKI can be a good predictor of the MMR of rectal adenocarcinoma.

The results of this study on the clinical data showed that the number of cases was higher in males than in females in the dMMR and pMMR group, and there was no statistical difference between the two groups in terms of gender and age between the two groups, which is inconsistent with the results of the study conducted by J.Toh et al. [29]. We analyzed that it may be caused by differences in ethnicity, geography, and dietary habits. Preoperative serum CA19-9 and CEA levels are now widely used in the diagnosis, staging and screening of rectal cancer [30]. The results of this study showed that there was no statistically difference in preoperative serum CA19-9 levels between the two groups, which may be attributed to the lower sensitivity and positivity of preoperative CA19-9 levels in the diagnosis of rectal adenocarcinoma [[31], [32], [33]]. Meanwhile, the level of preoperative CEA of dMMR group was significantly higher than the pMMR group. CEA is a glycoprotein that is produced by intestinal cancer tissues and acts as an antigen to trigger an immune response in patients. dMMR rectal adenocarcinomas have high tumour burden and active tumour growth, which can cause an increase in the preoperative serum CEA level. This finding is supported by the study conducted by Koyel et al. [34]. The pathology data revealed that the dMMR group exhibited a decreased incidence of vascular invasion and lymphatic node metastasis in comparison to the pMMR group. This disparity may be attributed to the fact that dMMR rectal adenocarcinoma tends to develop at an earlier stage and have less localized infiltration than the pMMR group [35].

Our results indicated that based on the whole tumor histogram analysis, the ADC could help to differentiate dMMR from pMMR under b-value of 1000 and 2000 s/mm^2^, and in the dMMR group all had lower ADC values than the pMMR group. Previous study has demonstrated that the existence of dMMR leads to an increase in the occurrence of shortened peptides caused by code-shifting mutations in cancer cells. This increase in immunogenicity stimulates the body's anti-tumor immune response, resulting in a higher number of infiltrating lymphocytes within the tumour. Consequently, the movement of water molecules between tumour cells becomes restricted [36]. The more compact the intercellular arrangement, the lower the ADC value, which may be the reason why the ADC value of rectal adenocarcinoma in the dMMR group is lower than the pMMR group.

In actual clinical work, considering the image signal-to-noise ratio and the existence of the T2 penetration effect, the selection of the b-value of the DWI sequence becomes particularly important, with the increase of the b-value, which is theoretically more conducive to the display of the lesion. Larger b-values should be chosen to measure the ADC value of the lesion under ideal conditions as the more accurate reflection of the water molecule movement, however, higher b-values can introduce non-Gaussian signals, contaminating the ADC measurement, while patients may not be able to tolerate the increased examination time. Therefore, in our study we used b-value of 1000 and 2000. Our results also showed that high b-value ADC value histogram analysis can help to differentiate rectal adenocarcinoma in dMMR and pMMR groups. Meanwhile, our results showed that the AUC values of ADCmean and ADC75th with b value of 2000 s/mm^2^ were about 0.728 and 0.713 in the dMMR group, and the AUC of skewness with b value of 1000 s/mm^2^ was a maximum of 0.818, which was different with the previous methods of generalizing ADC values (ADCmin and ADCmax), suggesting that ADC values can be further quantified to obtain more accurate imaging-biomarkers [37].

The results of this study showed that the D10th, D25th and D50th of the D-value in dMMR group were lower than the pMMR group. D value represents the diffusion ability of water molecules under non-Gaussian distribution; similar to ADC, it is a further derivation of ADC value. D value is lower with decreasing diffusion capacity of water molecules, which can more accurately reflect the actual diffusion situation of water molecules [38]. Rectal adenocarcinomas in the dMMR group had a lower D-value than those in the pMMR group, which can be better explained at the pathological level [39,40]. Meanwhile, the findings of this study indicated that K-values in the dMMR group were greater than the pMMR group, except for the K10th. The difference between the two groups was statistically significant at the K75th and K90th, with AUC values of 0.712 and 0.788, respectively. The K-value is used as the specific parameter of the DKI imaging, which is used to reflect the water molecules in biological tissues in the context of non-Gaussian distributed diffusive motions. Therefore, K-value can be considered as a quantitative indicator of the degree of heterogeneity of biological tissues [41,42]. Previous studies have indicated that DKI separates Gaussian and non-Gaussian diffusion effect, with MD representing diffusion and MK quantifying tissue complexity. Therefore, MK from DKI could effectively reflect tissue complexity (MK) in rectal adenocarcinoma [43,44]. This study demonstrates that dMMR tumours exhibit a pathological foundation of significant infiltration by lymphocytes, resulting in a densely organised tissue structure, elevated density of tumour cells, and a high tumour burden. Consequently, there is an increase in heterogeneity and the K values of dMMR tumours are higher compared to the pMMR group.

In the present study, the variables in which the difference between the dMMR group and the pMMR group for rectal adenocarcinoma was statistically significant were further analyzed by multivariate binary logistic regression analysis. It was found that ADC75th(b = 2000 s/mm^2^), ADCskewness (b = 1000 s/mm^2^) and Kskewness were the significant variables, with K skewness having the best diagnostic efficacy with an AUC value of 0.835. Skewness is a statistical measure that quantifies the degree of asymmetry in the probability distribution of a random variable. A higher skewness value indicates a pattern of data that is positively skewed, meaning that the distribution is skewed to the right of the mean. In such cases, the majority of the data is concentrated on the left side, and the median is smaller than the mean. We think this may be a reflection of the K75th and K90th are all effective in distinguishing MMR in rectal adenocarcinoma, which reflect the overall right side of the data, thus leading to an increase in skewness.

Our study still has some limitations. First, our center adopted a prospective approach to collect rectal occupancy cases, which reduced the selection bias to some extent. However, the limitations of the single-center study resulted in small sample size. Second, the lack of conventional sequence characteristics of MRI in the study may have certain limitations. Finally, the setting interval of b-value in DWI sequence scanning spanned a wide range and was not finely delineated.

In Conclusion, the whole tumor histogram parameters derived from DWI and DKI can effectively predict the MMR of rectal adenocarcinoma, indicating that these parameters can detailedly reflect the heterogeneity and the tumor microenvironment. In addition, the Kskewness parameter derived from the DKI was the most effective indicator to characterize the MMR of rectal adenocarcinoma.

## Data availability statement

Data will be made available on request.

## CRediT authorship contribution statement

**Hao Chen:** Writing – original draft, Methodology, Formal analysis. **Zhicheng Jin:** Writing – review & editing, Validation, Methodology. **Xiaoxiao Dai:** Validation, Resources. **Juan Zhu:** Writing – review & editing, Validation, Resources. **Guangqiang Chen:** Writing – review & editing, Formal analysis, Conceptualization.

## Declaration of competing interest

The authors declare that they have no known competing financial interests or personal relationships that could have appeared to influence the work reported in this paper.
